# Evidence for preexisting prion substrain diversity in a biologically cloned prion strain

**DOI:** 10.1371/journal.ppat.1011632

**Published:** 2023-09-05

**Authors:** Tess Gunnels, Ronald A. Shikiya, Taylor C. York, Alyssa J. Block, Jason C. Bartz

**Affiliations:** Department of Medical Microbiology and Immunology, Creighton University, Omaha, Nebraska, United States of America; University of Edinburgh, UNITED KINGDOM

## Abstract

Prion diseases are a group of inevitably fatal neurodegenerative disorders affecting numerous mammalian species, including Sapiens. Prions are composed of PrP^Sc^, the disease specific conformation of the host encoded prion protein. Prion strains are operationally defined as a heritable phenotype of disease under controlled transmission conditions. Treatment of rodents with anti-prion drugs results in the emergence of drug-resistant prion strains and suggest that prion strains are comprised of a dominant strain and substrains. While much experimental evidence is consistent with this hypothesis, direct observation of substrains has not been observed. Here we show that replication of the dominant strain is required for suppression of a substrain. Based on this observation we reasoned that selective reduction of the dominant strain may allow for emergence of substrains. Using a combination of biochemical methods to selectively reduce drowsy (DY) PrP^Sc^ from biologically-cloned DY transmissible mink encephalopathy (TME)-infected brain resulted in the emergence of strains with different properties than DY TME. The selection methods did not occur during prion formation, suggesting the substrains identified preexisted in the DY TME-infected brain. We show that DY TME is biologically stable, even under conditions of serial passage at high titer that can lead to strain breakdown. Substrains therefore can exist under conditions where the dominant strain does not allow for substrain emergence suggesting that substrains are a common feature of prions. This observation has mechanistic implications for prion strain evolution, drug resistance and interspecies transmission.

## Author summary

Prion strains operate as dynamic mixtures of a dominant strain and a minor population of substrains. Direct evidence, however, for the existence of preexisting substrains is lacking. Here we show that, using the well-characterized biologically cloned prion strain, DY TME, replication of the dominant strain is required to suppress replication of a minor, yet highly pathogenic substrain, HY TME. Using two complementary physical methods, we show that selective reduction of the dominant strain, in the absence of prion replication, allowed for the emergence of substrains. The identification of preexisting substrains may contribute to the ability of prions to rapidly adapt to new replication environments such as transmission to a new species or replication in the presence of anti-prion drugs.

## Introduction

Prion diseases are transmissible neurodegenerative disorders that affect mammals and are inevitably fatal. In humans, prion diseases include Creutzfeldt-Jakob disease (CJD), Gerstmann-Sträussler-Scheinker syndrome, fatal familial insomnia, and Kuru. Prion diseases in other animals are comprised of scrapie in sheep, bovine spongiform encephalopathy (BSE) in cattle, transmissible mink encephalopathy (TME) in ranch-raised mink, chronic wasting disease (CWD) in cervids, and camel prion disease. Prions can be zoonotic as evidenced by the interspecies transmission of BSE to humans resulting in the emergence of variant CJD [1,2,3]. CWD is an emerging prion disease that was first identified in Colorado in the 1960’s and is currently found in 30 US states, 4 Canadian provinces, South Korea and has recently been identified in Norway, Sweden, and Finland [4,5,6,7].

Prions are comprised of PrP^Sc^, the self-templating, disease-specific conformation of the host-encoded prion protein, PrP^C^ [8,9,10,11]. PrP^C^ is a glycosylphosphatidylinositol anchored cell surface protein with two N-linked glycosylation sites that is required for prion conversion and neurotoxicity [12,13,14,15,16,17]. Prion conversion occurs at the cell surface and/or in the endosomal lysosomal system resulting in a complete restructuring of PrP^C^ from a monomeric alpha helical structure to that of fibrillar parallel in-register intermolecular β-sheet (PIRIBS) structure [18,19,20,21]. Recent near-atomic resolution cryo-electron microscopy (EM) studies have also provided important structural evidence for the interaction of PrP^Sc^ with polyanionic cellular cofactors that facilitate prion conversion [20,21].

Prion strains are operationally defined by heritable differences in the phenotype of disease upon defined transmission conditions [22]. The prion strain-specific phenotype of disease can include incubation period, clinical signs of infection, strain mutation rate, tropism of prion conversion within and between tissues and zoonotic potential [23,24,25,26,27,28,29,30,31,32,33]. Strain-specific differences in the biochemical features of PrP^Sc^ include migration on SDS-PAGE following proteinase K (PK) digestion, conformational stability in chaotropic agents and *in vitro* conversion efficiency [34,35,36,37,38]. These biochemical features of PrP^Sc^ are consistent with the hypothesis that strain-specific conformations of PrP^Sc^ encode prion strain diversity [34,39]. Cryo-EM analysis of PrP^Sc^ of the murine-adapted scrapie strains RML and ME7 indicate that while they both share PIRIBS architecture, there are strain-specific differences in the subfolding of PrP rungs providing the most direct evidence to date in support of this hypothesis [21,40].

Prions exist as mixtures of strains. Scrapie-infected sheep and patients with sporadic CJD can contain mixtures of prion strains as determined by strain-specific Western blot migration profiles of PrP^Sc^ [41,42,43,44,45]. Passage of these field isolates to transgenic mice expressing either ovine or human PrP^C^ can result in the isolation of distinct prion strains consistent with the hypothesis that an individual can be simultaneously infected with more than one prion strain [42,46]. Experimental inoculation of rodents with more than one prion strain indicates that prion strains can compete for PrP^C^ and that the dominant strain can suppress, but not eliminate, the minor strain [47,48,49,50]. Interestingly, treatment of rodents with anti-prion therapies can result in the emergence of drug-resistant prion strains that revert to a drug sensitive state following removal of the anti-prion drug [51,52,53,54,55,56,57]. These observations are consistent with the hypothesis that prion strains are comprised of a dominant strain and substrains [58,59,60]. While a wealth of experimental evidence supports this hypothesis, direct observation of substrains has not been documented. Here we investigated if the well-characterized biologically cloned drowsy (DY) strain of hamster-adapted TME contained substrains.

## Results

### Suppression of HY replication by DY TME *in vitro*

**Fig 1:**
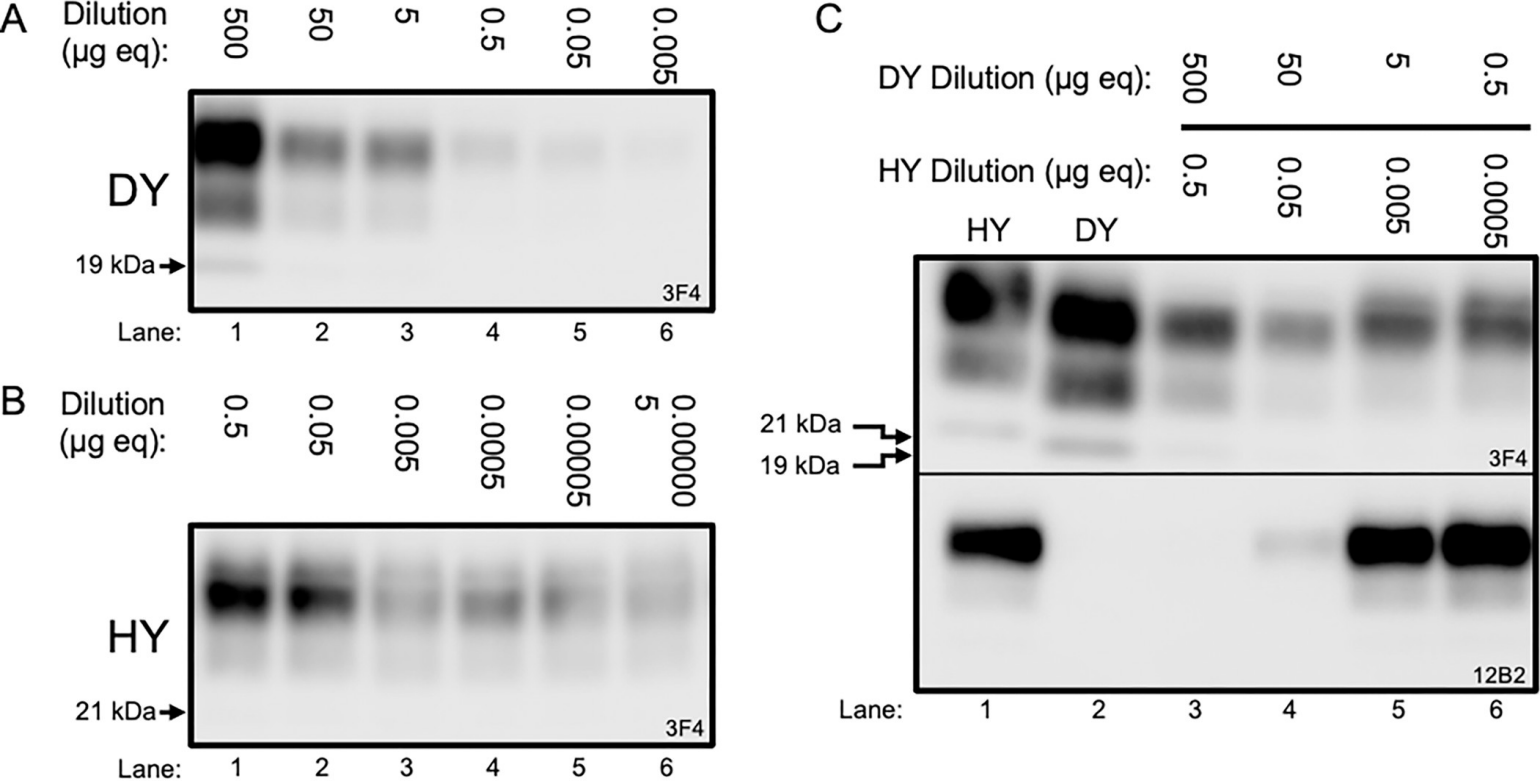
Suppression of HY PrP^Sc^ formation by DY TME. (A-C) Western blot analysis of representative PMCA serial dilution samples (n = 3) of DY PrP^Sc^ alone (A), HY PrP^Sc^ alone (B) and mixtures of HY and DY PrP^Sc^ (C). Input samples were ten-fold serially diluted, subjected to one round of PMCA followed by PK digestion, and probed by immunoblotting using antibodies 3F4 (detects all strains) and 12B2 (specific for an epitope present on HY PrP^Sc^ but not DY PrP^Sc^). Both strains amplify independently, with HY PrP^Sc^ having higher replication efficiency compared to DY PrP^Sc^. When mixed at a constant HY to DY ratio of 1:1000, the 500:0.5 μg eq mixture shows complete suppression of HY PrP^Sc^ amplification, while the 50:0.05 μg eq. mixture shows incomplete suppression, with some HY PrP^Sc^ being detectable in the sample using the 12B2 antibody. When DY PrP^Sc^ is below 50 μg eq, HY PrP^Sc^ amplifies without interference.

Protein misfolding cyclic amplification (PMCA) reactions seeded with 10-fold serial dilutions of either DY or HY PrP^Sc^ separately reveals differential amplification efficiency between the two strains while maintaining their strain-specific migration pattern of 19- and 21-kDa, respectively (Fig 1, panels A and B). To investigate prion strain interference during prion co-infection, serial 10-fold dilutions of a 1000:1 ratio DY to HY TME were seeded into PMCA reactions (Fig 1, panel C). Following one round of PMCA, DY TME suppressed the replication of HY TME as evidenced by migration of PrP^Sc^ and immunoreactivity to the anti-PrP antibody 12B2 that recognizes HY but not DY PrP^Sc^ (S1 Fig). Strain interference was observed under concentrations where robust DY PrP^Sc^ amplification was detected (500:0.5 μg eq and 50:0.05 μg eq DY to HY; Fig 1, panel C, lanes 3 and 4). As replication of DY PrP^Sc^ diminishes following serial dilution a corresponding increase in HY PrP^Sc^ was detected as evidenced by the migration of PrP^Sc^ and the emergence of 12B2 immunoreactive PrP^Sc^ (Fig 1, panel C, lanes 4–6). Overall, these data suggest that DY PrP^Sc^ replication suppresses HY PrP^Sc^ formation.

### DY TME is not a class III prion strain

Biological stability of prion strains ranges from class I strains being highly stable to class III strains breaking down to a shorter incubation period strain [61]. Breakdown of class III strains occurs more frequently when passaged at high prion titer compared to low titer [27,28]. We routinely passage DY TME inoculum at low titer (>10^−4^ dilution of brain homogenate) and have not observed changes in the strain properties of DY TME [32,37,62,63,64,65,66]. To more rigorously investigate if DY TME is a class III strain, we serially passaged DY TME at high titer (10^−1^ dilution of brain homogenate) by the intracerebral (i.c.) route of infection for five serial passages. Each serial passage of DY TME was accompanied with an uninfected negative control group. In all (n = 5) of the animals for each serial passage, the DY TME-infected animals maintained clinical signs, incubation period, PrP^Sc^ migration and guanidine hydrochloride (Gdn-HCl) conformational stability properties of DY TME (Table 1). None (n = 5) of the negative control group animals included for each serial passage developed clinical signs of prion infection by 250 days post infection (dpi). DY TME is not lymphotropic and does not cause infection by extraneural routes of infection [32,63]. To investigate if lymphotropic strains are present in the 4^th^ i.c. serial high titer hamster passage of DY TME-infected brain, groups (n = 5) of hamsters were inoculated by either the intraperitoneal (i.p.) or extranasal (e.n.) routes of infection. None (n = 5) of the DY TME or uninfected negative control group animals i.p. or e.n. inoculated developed clinical signs of prion infection by 650 dpi (Table 1 and S2 Fig). Overall, these data indicate that DY TME is a stable prion strain.

**Table 1:** Serial high-titer passage of DY TME results in the retention of DY TME strain properties.

						PrP^Sc^ properties	
Inoculum	Serial passage	Inoc. route^a^	Inc. period^b^	Attack rate^c^	Clinical signs	Migration	Conf. stability^e^
DY 10^−2^	-	i.c.	181±6	10/10	PL	19 kDa	1.83±0.03 (n = 12)
DY 10^−1^	1	i.c.	159±3	5/5	PL	19 kDa	1.73±0.04 (n = 12)
DY 10^−1^	2	i.c.	159±3	5/5	PL	19 kDa	1.71±0.05 (n = 12)
DY 10^−1^	3	i.c.	161±6	5/5	PL	19 kDa	1.81±0.03 (n = 12)
DY 10^−1^	4	i.c.	154±3	5/5	PL	19 kDa	1.87±0.03 (n = 12)
DY 10^−1^	5	i.c.	170±4	4/5^d^	PL	19 kDa	n.d.
DY 10^−1^	5	i.p	>650	0/5	n.a.	n.a.	n.a.
DY 10^−1^	5	e.n.	>650	0/5	n.a.	n.a.	n.a.

^a^ i.c.–intracerebral; i.p–intraperitoneal; e.n.—extranasal

^b^ days post infection±SEM

^c^ number inoculated / number affected

^d^ one intercurrent death at 102 dpi

^e^ [Gdn-HCl]_1/2_

n.d.–not done

n.a.–not applicable

PL–progressive lethargy

### Detection of PrP^Sc^ substrains in DY TME-infected brain

**Fig 2:**
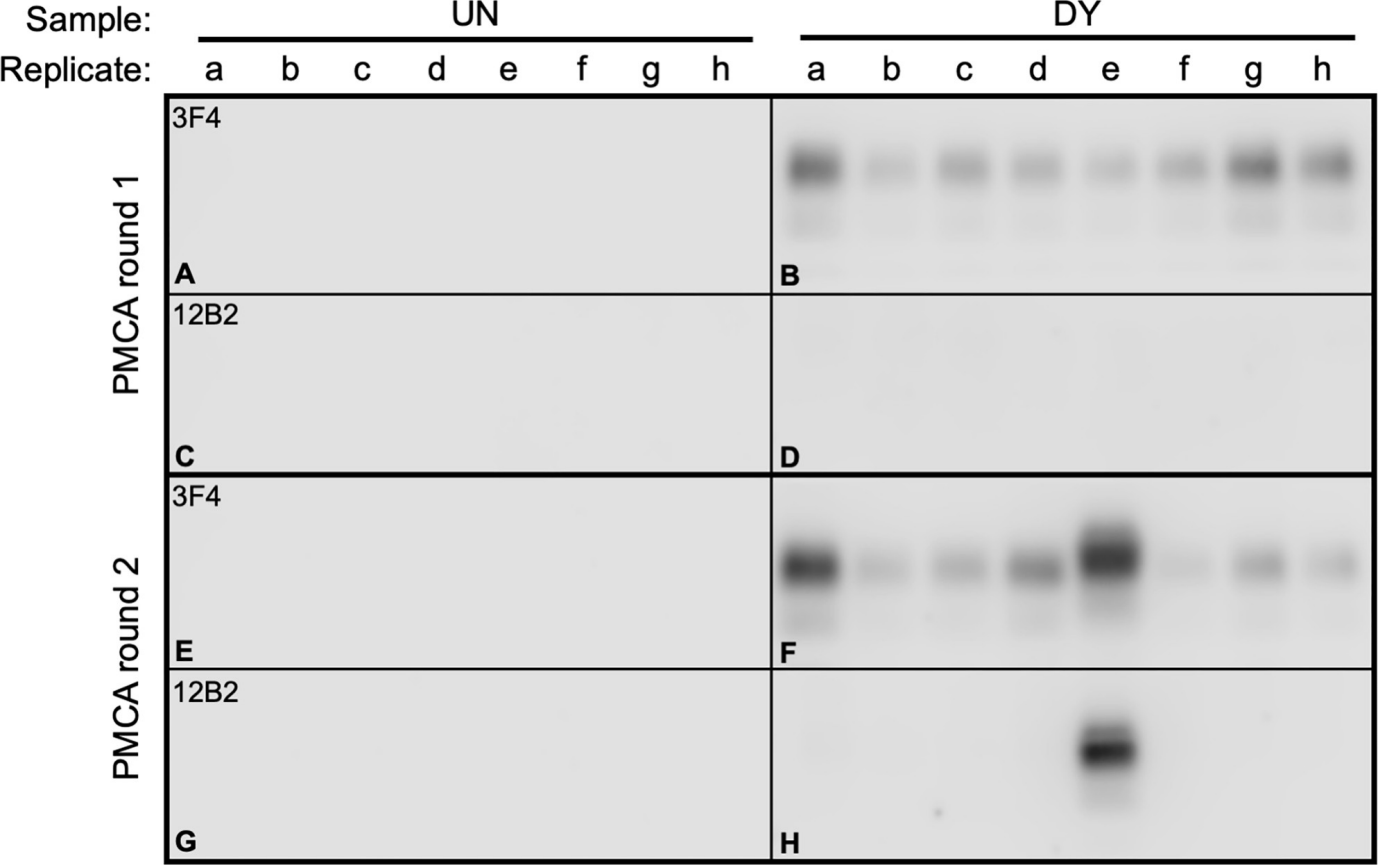
Extended PK digestion of DY TME-infected brain homogenate reveals the presence of non-DY PrP^Sc^ species. Western blot analysis of proteinase K strain selection assay products seeded with uninfected (UN; panel A,C,E,G) or drowsy (DY) brain homogenate (panels B, D, F, H) after one (panels A-D) or two (panels E-H) rounds of PMCA probed with either the anti-PrP antibody 3F4 (panels A-B, E-F) or 12B2 (panels C-D, G-H).

DY can suppress replication of short incubation period, highly pathogenic strains (Fig 1). DY PrP^Sc^ is more susceptible to digestion with proteinase K (PK) compared to other known hamster prion strains (S3 Fig) [34,37,67]. We reasoned that extended PK digestion of DY TME-infected brain homogenate would reduce the suppressive pressure of the dominant strain that may allow for detection of PrP^Sc^ from substrains with relatively higher PK resistance. Uninfected brain homogenate was subjected to the proteinase strain selection assay (PSSA) did not result in PMCA detection of PrP^Sc^ (Fig 2, panels A, C, E, G). PMCA reactions seeded with PSSA of biologically-cloned DY-infected brain homogenate resulted in detection of PrP^Sc^ that was immunoreactive with 3F4 in both first and second serial round of PMCA (Fig 2, panel B and F, respectively) and was immunoreactive to 12B2 only in a subset of reactions following the second serial round of PMCA (Fig 2, panel H, lanes e). Non-PK digested uninfected or DY TME brain homogenate seeded PMCA reactions either failed to amplify PrP^Sc^ or maintained DY PrP^Sc^ properties, respectively (S4 Fig). This pattern of 3F4 and 12B2 immunoreactivity is inconsistent with the DY PrP^Sc^ that was added to the PSSA reaction suggesting it is a non-DY conformation of PrP^Sc^. [37] (S1 Fig).

**Fig 3:**
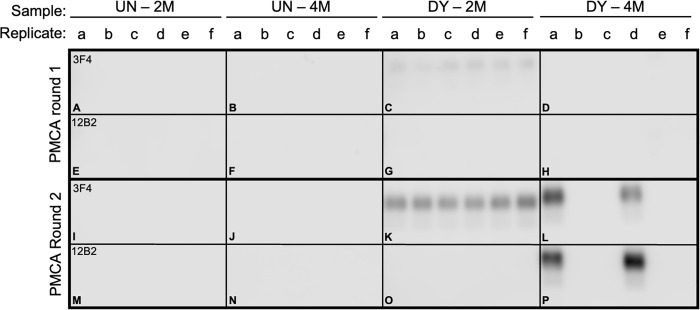
Detection of prion substrains in DY TME-infected brain. Western blot analysis of conformational strain selection assay products seeded with uninfected (UN; panel A,B,E,F,I,J,M,N) or drowsy (DY) brain homogenate at either 2M (panels C,G,K,O) or 4M (panels D,H,L,P) Gdn-HCl after one (panels A-H) or two (panels I-P) rounds of PMCA probed with either the anti-PrP antibody 3F4 (panels A-D, I-L) or 12B2 (panels E-H, M-P).

The conformational stability of DY PrP^Sc^ is lower compared to other hamster-adapted prion strains [37,38]. Based on this observation, we reasoned that denaturation and degradation of the relatively low conformational stability DY PrP^Sc^ could reduce the suppressive pressure of the dominant strain and allow for interrogation of the sample for substrains with higher PrP^Sc^ conformational stabilities that are below the limit of Western blot detection by using PMCA. Uninfected brain homogenate that was subjected to the conformational strain selection assay (CSSA) at either 2M or 4M Gdn-HCl did not result in PMCA detection of PrP^Sc^ (Fig 3, panels A, B, E, F, I, J, M, N). CSSA reactions seeded with DY-infected brain homogenate treated at 2M Gdn-HCl resulted in PrP^Sc^ that was immunoreactive with 3F4 in both PMCA round 1 (Fig 3, panel C) and round 2 (Fig 3, panel K) in all (n = 6) of the replicates but was not immunoreactive with 12B2 (Fig 3, panels G, O) consistent with DY PrP^Sc^ (S1 Fig). CSSA reactions seeded with DY TME-infected brain homogenate treated at 4M Gdn-HCl did not result in detectable PrP^Sc^ following one round of PMCA with either 3F4 or 12B2 (Fig 3, panels D, H). However, upon second serial round of PMCA, PrP^Sc^ was detected in a subset of replicates that was immunoreactive with both 3F4 and 12B2 (Fig 3, panels L, P). This pattern of PrP^Sc^ immunoreactivity is inconsistent with the DY PrP^Sc^ that was added to the CSSA reaction and instead is consistent with PrP^Sc^ from other hamster-adapted strains [37] (S1 Fig). Non-PK digested uninfected or DY TME brain homogenate seeded PMCA reactions either failed to amplify PrP^Sc^ or maintained DY PrP^Sc^ properties, respectively (S4 Fig). Overall, using two different experimental approaches, we have identified a relatively low abundance PrP^Sc^ subpopulation with PrP^Sc^ properties distinct from the dominant parental strain, DY TME.

### Hamsters infected with CSSA products have a bona fide prion infection

**Fig 4:**
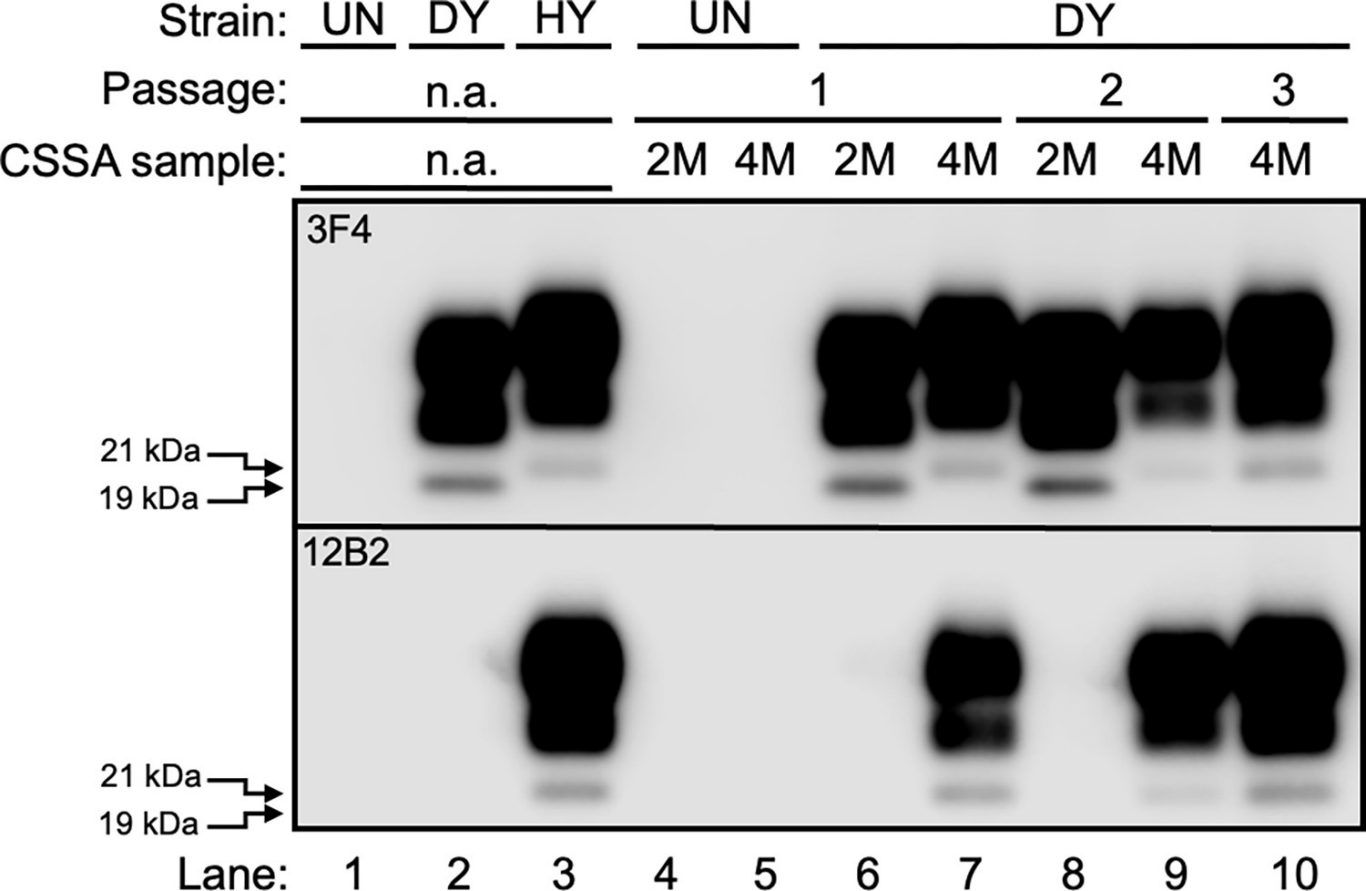
Hamsters infected with CSSA products have a bona fide prion infection. Western blot analysis of proteinase K digested brain homogenate from mock infected hamster (UN; lane 1), DY TME infected hamster (DY; lane 2), HY TME infected hamster (HY; lane 3) or CSSA products from mock-infected reactions (lanes 4 and 5) or DY CSSA reactions with either 2M (lane 6) or 4M (lane 7) Gdn-HCl. Second (lanes 8 and 9) and third (lane 10) serial hamster passage of brain material from hamsters infected with CSSA products from lanes 6 and 7. Western blots were probed with either the anti-PrP antibody 3F4 (top panel) that recognizes both the 19 (lane 2) and 21 kDa (lane 3) unglycosylated PrP^Sc^ polypeptide or the anti-PrP antibody 12B2 which recognizes the 21 kDa (lane 3) but not the 19 kDa (lane 2) unglycosylated PrP^Sc^ polypeptide. The migration of the 19 and 21 kDa unglycosylated PrP^Sc^ polypeptide are indicated at the left of the panel.

Hamsters were inoculated with the products of the CSSA to determine if they were infectious. All (n = 5) hamsters i.c. inoculated with either 2 or 4M Gdn-HCl uninfected CSSA reactions failed to cause disease by 280 dpi (Table 2) and did not contain detectable PrP^Sc^ in PK-digested brain homogenates (Fig 4, lanes 4 and 5). All (n = 4, one intercurrent death at 191 dpi) hamsters inoculated with second round PMCA reaction from 2M Gdn-HCl DY TME seeded CSSA reactions (Fig 3, panel H, K, replicate d) developed clinical signs of progressive lethargy at 214±5 dpi and contained PrP^Sc^ that was immunoreactive with the anti-PrP antibody 3F4 (Fig 4, lane 6, top panel) with a 19 kDa migration of the unglycosylated PrP^Sc^ polypeptide. The anti-PrP antibody 12B2 failed to detect PrP^Sc^ from this sample (Fig 4, lane 6, bottom panel). A second serial hamster passage of this brain homogenate resulted in all (n = 5) of the hamsters developing clinical signs of progressive lethargy at 174±3 dpi (Table 2) with these animals maintaining the PrP^Sc^ immunoreactivity and migration properties from first hamster passage (Fig 4, lane 8). Hamsters inoculated with second round PMCA reaction from a 4M Gdn-HCl DY TME seeded CSSA reaction (Fig 3, panel I, L replicate d) developed clinical signs of hyperexcitability at 91±3 dpi (Table 2) and contained PrP^Sc^ that was immunoreactive with both the anti-PrP antibodies 3F4 (Fig 4, lane 7, top panel) and 12B2 (Fig 4, lane 7, bottom panel) with a 21 kDa migration of the unglycosylated PrP^Sc^ polypeptide. Second and third serial hamster passage of this brain homogenate resulted in all (n = 5) hamsters developing clinical signs of hyperexcitability at 65±3 and 59±3 dpi, respectively, and retained the PrP^Sc^ immunoreactivity and migration patterns from first hamster passage (Fig 4, lanes 9 and 10). All (n = 5) groups of mock-infected controls included for second and third hamster passage remained clinically normal by 250 dpi (S5 Fig and S1 Table). Overall, the CSSA products are infectious, and the properties of the hamsters infected with the 2M DY TME CSSA products are consistent with infection with DY TME. In contrast, hamsters infected with the 4M DY TME CSSA products have clinical signs, incubation periods and PrP^Sc^ Western blot migration properties that differ from the parental strain, DY TME.

**Table 2:** Transmission and adaptation of hamster substrains.

	Hamster passage number					
	First		Second		Third	
Inoculum	Inc. Period^a^	Clinical	Inc. Period	Clinical	Inc. Period	Clinical
UN 2M CSSA	≥280 (0/5)	n.a.	n.d.	n.a.	n.d.	n.a.
UN 4M CSSA	≥280 (0/5)	n.a.	n.d.	n.a.	n.d.	n.a.
DY 2M CSSA	214±5 (4/5)^b^	PL	174±3 (5/5)	PL	n.d.	n.a.
DY 4M CSSA	91±3 (5/5)	HA	65±3 (5/5)	HA	59±3 (5/5)	HA
UN b.h.	≥225 (0/5)	n.a.	n.d.	n.a.	n.d.	n.a.
DY b.h.	169±4 (5/5)	PL	n.d.	n.a.	n.d.	n.a.
HY b.h.	60±3 (5/5)	HA	n.d.	n.a.	n.d.	n.a.

^a^ Days±SEM (number affected / number inoculated)

^b^ intercurrent death at 191 dpi

n.a.–not applicable

n.d.–not done

b.h.–brain homogenate

PL–progressive lethargy

HA–Hyperexcitability and ataxia

### Conformational stability of DY TME substrain PrP^Sc^ is consistent with the CSSA selection criteria

**Fig 5:**
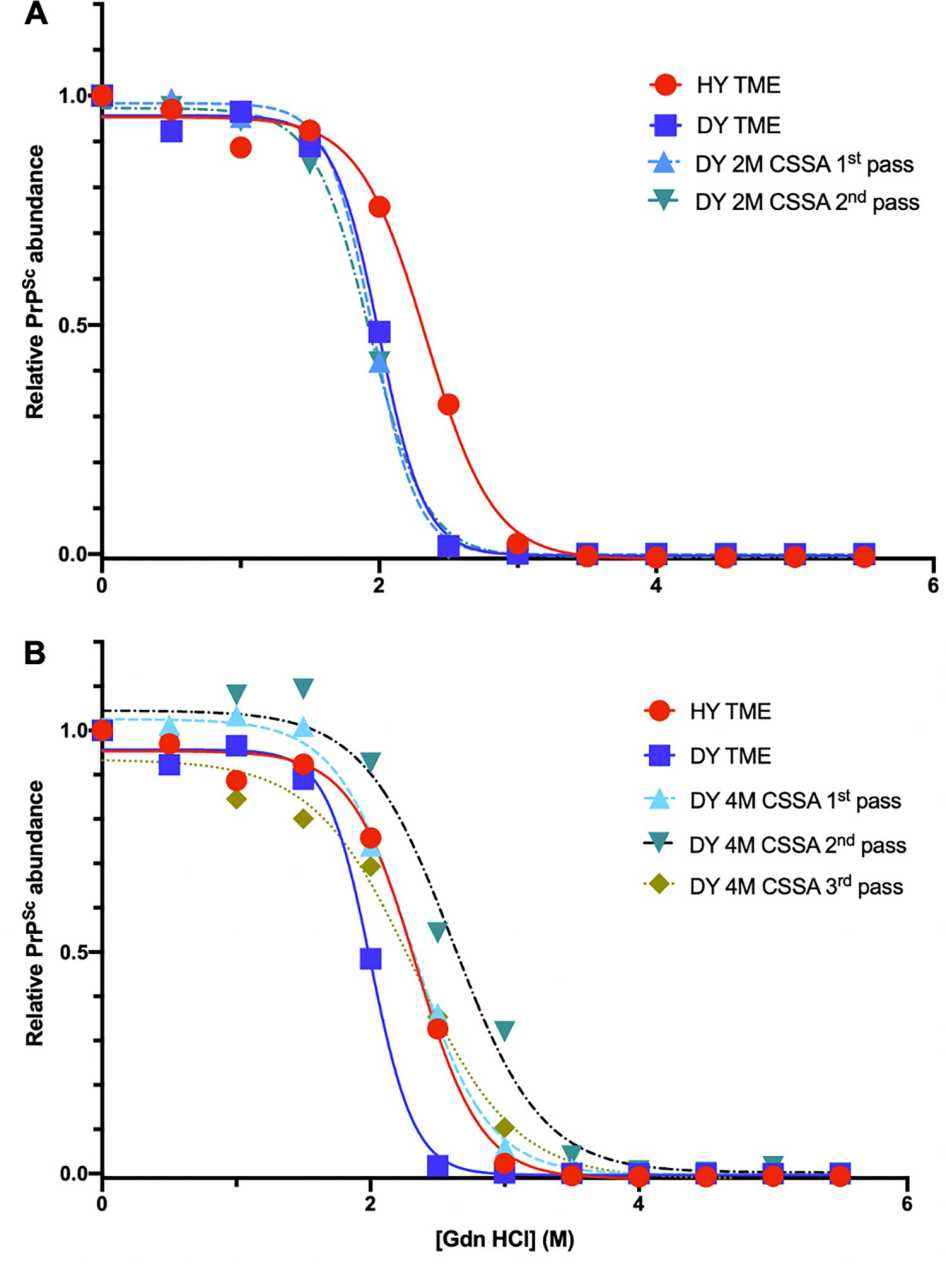
Conformational stability of PrP^Sc^ from hamsters infected with brain-derived prion strains and the CSSA isolated substrain differ. Representative PrP^Sc^ conformational stability curves from hamsters infected with either HY TME, DY TME, 2M DY CSSA reaction products (panel A), or 4M DY CSSA reaction products (panel B). The conformational stability curves were repeated a minimum of 8 times with similar results.

The [Gdn-HCl]_1/2_ value of PrP^Sc^ from brain homogenates from HY or DY TME-infected hamsters was 2.33±0.02 (n = 16) and 1.95±0.01 (n = 34), respectively (Fig 5 and S1 Table). The [Gdn-HCl]_1/2_ value of PrP^Sc^ from brain homogenates of hamsters inoculated with either 2M DY TME CSSA reaction products or 1^st^ hamster passage of 2M DY TME CSSA reaction products was 2.05±0.04 (n = 8) and 1.94±0.02 (n = 16), respectively (Fig 5, panel A and S1 Table) and did not significantly (p>0.05) differ compared to DY TME. The [Gdn-HCl]_1/2_ value of PrP^Sc^ from brain homogenates of hamsters inoculated with either 4M DY TME CSSA reaction products, 1^st^ hamster or 2^nd^ hamster passage of 4M TME CSSA reaction products was 2.46±0.07 (n = 13), 2.57±0.03 (n = 30), and 2.34±0.04 (n = 16), respectively (Fig 5, panel B and S1 Table). The [Gdn-HCl]_1/2_ value of first and second hamster passage significantly differed compared to HY TME (p<0.05), while the third passage value did not significantly (p>0.05) differ compared to HY TME. Overall, the [Gdn-HCl]_1/2_ value of hamsters infected with 2M DY TME CSSA reaction products was consistent with infection with DY TME, while hamsters infected with 4M DY TME CSSA reaction products had [Gdn-HCl]_1/2_ values that were higher than HY TME on first and second hamster passage that, by third hamster passage, were similar to HY TME-infected animals.

## Discussion

Replication of the dominant prion strain can suppress replication of prion substrains. It is known that when two prion strains infect the same host one strain can interfere with or completely block another strain from causing disease [32,47,68,69]. The relative onset of prion replication between the two strains, in a common population of cells, dictates which strain will emerge [49,50,70]. Altering either the relative ratios of the two strains that are infected at the same time (co-infection), or the time interval between inoculation of the first and second prion strain (superinfection) will determine which strain emerges [48,66]. Mechanistically, strains compete for PrP^C^, however, it is unclear if the blocking strain PrP^Sc^ simply binds to PrP^C^ rendering it inaccessible for the other strain (site blocking) or if prion replication is required for strain interference to occur [25,49,50,70]. To discriminate between these two possibilities, we used a ratio of DY and HY where DY can block HY from emerging in PMCA and 10-fold serial dilutions of the DY and HY mixture were subjected to PMCA. This experimental approach keeps the ratio of DY and HY PrP^Sc^ the same in all dilutions tested but since DY has a lower PMCA conversion activity per unit PrP^Sc^ compared to HY, as the strain mixture is diluted, DY conversion is reduced at a proportionally faster rate [37]. We found that the ability of DY to interfere with HY was strong when DY conversion is robust, but, as DY conversion decreased, HY was able to emerge despite having the same ratio of DY to HY PrP^Sc^ (Fig 1). Based on this observation, we hypothesize that DY conversion may contribute to the strain interference effect.

Prions exhibit properties of quasispecies. Treatment of rodents with anti-prion therapies can result in the emergence of drug-resistant prion strains and subsequent removal of the anti-prion drug results in reversion to a drug-sensitive state [51,52,53,54,55,56,57]. Serial repeated passage of prions at low titer (i.e., bottlenecking) results in a decrease in prion fitness [71]. This observation is consistent with Muller’s ratchet, where populations with a high mutation rate (i.e., quasispecies) undergo a reduction in fitness during bottlenecking events [72,73,74]. These observations led to the hypothesis that prions are quasispecies; a population of similar, but not identical conformations of PrP^Sc^ [58,59,74]. The emergence of drug-resistant prions is hypothesized to be the result of the suppression of the dominant strain by the anti-prion therapy allowing for the emergence of a preexisting drug resistant substrain, analogous to what occurs in conventional microorganisms [75]. It is unclear, however, if the treatments select for a preexisting substrain or, alternatively, change the conformation of PrP^Sc^ during prion formation comparable to what has been observed with prion conversion cofactors [76]. While the existence of prion substrains is supported by much evidence, direct observation of substrains has not been documented.

Prions are comprised of a dominant strain and substrains. Building upon our observation that PK digestion of a mixture of DY and HY allows for a more rapid emergence of HY PrP^Sc^ [77], we found that extended PK digestion of DY TME resulted in the amplification of PrP^Sc^ with different biochemical properties compared to the parental strain, DY TME (Fig 2). Since PK digestion does not change strain properties and is independent of prion conversion, we interpret this finding as evidence of a preexisting substrain [78]. The conformational stability of PrP^Sc^ is strain specific [36,37] and we reasoned that denaturation and PK digestion of relatively low conformational stability PrP^Sc^ would reduce the suppressive pressure of the dominant strain, allowing for the emergence of substrains with relatively higher PrP^Sc^ conformational stabilities. Uninfected brain homogenate that was subjected to the conformational strain selection assay (CSSA) at either 2M or 4M Gdn-HCl did not result in detection of PrP^Sc^ or prion infectivity (Fig 3, panels A, B, E, F, I, J, M, N; Table 1), indicating that PrP^Sc^ was not introduced into the CSSA reaction either via exogenous sources (e.g., contamination) or by *de novo* prion formation by the process itself. DY TME seeded 2M CSSA reactions resulted in detection of DY PrP^Sc^ that, upon passage into hamsters, had an incubation period, clinical signs, PrP^Sc^ migration and conformational stability properties of DY TME (Tables 2 and S1 and Figs 4 and 5). Taken together, these data suggest that in the 2M DY TME Gdn-HCl CSSA reactions, DY PrP^Sc^ abundance is reduced, but not to a sufficient level to allow for the emergence of substrains. As transmission of this material to hamsters results in the maintenance of DY TME strain characteristics, this indicates that the CSSA assay and subsequent PMCA is not modifying DY TME strain properties. This is consistent with previous studies where treatment of prion strains with Gdn-HCl altered infectivity, but not the prion strain [79,80,81] and PMCA generated prions maintain the properties of the strain they are seeded with [49,70]. CSSA reactions seeded with DY TME-infected brain homogenate treated at 4M Gdn-HCl resulted in detection of PrP^Sc^ only after the second round of PMCA in subset of replicates that was immunoreactive with both 3F4 and 12B2 (Fig 3, panels L, P, replicates a and d). These observations suggest the treatment conditions in the 4M DY TME CSSA reactions reduced the suppressive effect of DY PrP^Sc^ sufficiently to allow for detection of substrains present in the DY TME-infected brain. The selection methodology occurred in the absence of prion formation; therefore, we hypothesize that the substrains are preexisting. Transmission of this material to hamsters resulted in the development of clinical signs of hyperexcitability, PrP^Sc^ that was immunoreactive with both of the anti-PrP antibodies 3F4 (Fig 4, lane 7, top panel) and 12B2 (Fig 4, lane 7, bottom panel) and PrP^Sc^ with conformational stability higher relative to other known hamster prion strains [37,38] (Fig 5 and Table 2). These observations suggest that this is a preexisting substrain present in the DY TME-infected brain homogenate with properties consistent with the selection criteria (i.e., relatively higher PrP^Sc^ conformational stability) and not contamination (Table 2). Second and third serial hamster passage of the 4M DY TME CSSA material resulted in a shortening of the incubation period, maintenance of the clinical signs and PrP^Sc^ electrophoretic mobility and 12B2 immunoreactivity (Table 2 and Fig 4). Interestingly, by third hamster passage the PrP^Sc^ conformational stability was comparable to that of short incubation period strains in hamsters [37,38] (Table 2).

The overrepresentation of prion strains with similar properties from diverse transmission histories has long been observed [35,82,83]. In hamsters, short incubation period, high PrP^Sc^ conformational stability strains with clinical signs of hyperexcitability and ataxia have been isolated following the interspecies transmission of TME, scrapie and CWD [35,66,82,83]. In mice, the ME7 strain was isolated in approximately over half of the mice inoculated with various sources of sheep scrapie [84,85,86]. It is hypothesized that a given primary amino acid sequence of PrP will have a thermodynamically favored conformation (e.g. strain) of PrP^Sc^ [58]. The transmission history of the 4M DY TME CSSA product suggests that this material contained a mixture of strains that, upon serial passage in hamsters, evolved to a strain with PrP^Sc^ properties resembling other overrepresented short-incubation period hamster strains consistent with this hypothesis.

We hypothesize that substrains are a common feature of prion strains. DY TME is biologically stable and not prone to strain breakdown. The identification of substrains in DY TME-infected brain suggests that substrains can exist under conditions where the dominant strain does not allow for substrain emergence. The two complementary methodologies for substrain identification allowed for exploration of only a portion of the possible substrain repertoire and restricted the properties of the substrains that could be identified. Additionally, PMCA may only identify a subpopulations of existing strains whereas a newly described method of PMCA utilizing shaking in place of sonication can identify metastable PrP^Sc^ conformations [87]. Despite the bias in strain selection and PMCA, substrains were identified and we hypothesize that the diversity of substrains is much greater than what is reported here. Overall, these findings provide important mechanistic insight into prion strain biology, the selection of drug resistant prion strains, and interspecies transmission.

## Materials and methods

### Ethics statement

All procedures involving animals were approved and in compliance with the Guide for the Care and Use of Laboratory Animals (protocol numbers 880 and 1030) by the Creighton University Institutional Animal Care and Use Committee.

### Prion strains

Prion strains are maintained by intracerebral passage (i.c.) at a 10^−4^ or greater dilution of brain homogenate. Brains from terminally-ill hamsters inoculated with either the HY (10^9.3^ i.c. LD_50_/g) or DY (10^7.4^ i.c. LD_50_/g) biologically-cloned strains of hamster-adapted TME [88] were homogenized to 10% w/v in Dulbecco’s phosphate buffered saline (DPBS) (Mediatech, Herndon, VA) using disposable syringes, needles and plasticware. All homogenates were stored at -80°C.

### Animal bioassay

Male Syrian hamsters (Harlan-Sprague-Dawley, Indianapolis, IN) were i.c. inoculated with 25 μl of either a 1% w/v brain homogenate or a 1:10 dilution of PMCA generated material in DPBS. Hamsters were observed three times per week for the onset of clinical signs of prion disease and the incubation period was calculated as the number of days between inoculation and onset of clinical signs. Two tail Student’s T test (Prism Version 8.4.3, for Mac; GraphPad Software Inc., La Jolla, CA) with a p value of 0.01 was used to compare incubation periods. All tissues were collected with strain dedicated tools that are decontaminated between animals by immersion in bleach (neat) for 15 minutes at room temperature.

### Conformational stability assay

The PrP^Sc^ conformational stability assay was performed as described previously [89]. Briefly, brain homogenate (1% w/v) was incubated in Gdn-HCl (Sigma-Aldrich, St. Louis, MO) ranging from 0 M to 3.5 M while shaking for one hour at room temperature. The concentration of Gdn-HCl was adjusted to 0.5 M prior to transferring to a 96-well filter plate with a PVDF membrane bottom (Merck Millipore, Co. Cork, Ireland). Samples were digested with PK (5 μg/mL; 1:100 PK:BH) for one hour at 37°C (5 μg/ml; Roche Diagnostics, Mannheim, Germany) followed by incubation with phenylmethane sulfonyl fluoride (PMSF; MP Biomedicals, LLC, Salon, OH) for 20 minutes at room temperature. Endogenous peroxidases were inhibited with 0.3% H_2_O_2_ in methanol and the PVDF membrane blocked using 5% w/v nonfat dry milk in TTBS (BioRad Laboratories, Hercules, CA). The hamster prion protein was immunodetected using the mouse monoclonal anti-PrP antibody 3F4 (final concentration of 0.1 μg/mL; EMD Millipore, Billerica, MA). The membrane was developed with the Pierce SuperSignal West Femto system (Pierce, Rockford, IL) and imaged on a Li-Cor Odyssey Fc Imager (Li-Cor, Lincoln, NE). PrP^Sc^ signal intensity was determined using Li-cor Image Studio Software v.5.2.5 (Lincoln, NE). The point where half of PrP^Sc^ is in a PK resistant state and half is in a PK sensitive state (i.e. [Gdn-HCl]_1/2_) was determined by calculating the log IC_50_ of the non-linear curve fitted to the normalized data (GraphPad Software, San Diego, CA). PrP^Sc^ denaturation curves were generated using GraphPad Prism (GraphPad Software, San Diego, CA). Statistical comparison of the [GdnHCl]_1/2_ values were performed using Student’s t-test (GraphPad Software, San Diego, CA).

### Proteinase strain selection assay

250 μl of 10% brain homogenate is digested at 37°C for 24 hours with 250 μl of 200 μg/ml proteinase K solution. (Roche Diagnostics, Mannheim, Germany). To remove PK from the sample prior to PMCA, the PK digested brain homogenate is incubated at 37°C for 1 hour with 1 μl benzonase (MilliporeSigma, Burlington, MA). The sample is then incubated at room temperature for one hour with 250 μl of sarkosyl solution (20% N-lauroylsarcosine in 10 mM Tris Buffer pH 7.5), 1 μl DL-dithiothreitol 250 mM (Sigma-Aldrich, Burlington, MA), and 1 μl of Antifoam (Sigma-Aldrich, Burlington, MA). After incubation, the sample is centrifuged at 10,000 x g for 30 minutes, the pellet is discarded, and the supernatant centrifuged at 100,000 x g for 1 hour. The supernatant is discarded, the pellet resuspended in DPBS (Corning, Corning, NY) and centrifuged at 100,000 x g for 1 hour. The supernatant is discarded, the pellet resuspended in 0.1% sarkosyl solution (0.1% N-lauroylsarcosine in DPBS) and stored at -80°C.

### Conformational strain selection assay

10% w/v brain homogenate is diluted in detergent buffer (5% sodium deoxycholate and 5% Igepal in Dulbecco’s Phosphate Buffered Saline [(DPBS), Corning, Corning, NY] and centrifuged at 15,000 x g for 5 minutes. Supernatant is collected and the pellet discarded. 20 μl of the supernatant is treated with increasing concentrations of Gdn-HCl (Millipore Sigma, Burlington, MA) (0M, 2M, or 4M) at room temperature for 2 hours. Each sample tube is normalized to 0.5M Gdn-HCl prior to digestion with 20 μg/ml of PK (Roche Diagnostics, Mannheim, Germany) for 1 hour at 37°C. The PK digestion is stopped with 2 mM phenylmethane sulfonyl fluoride (PMSF; Millipore Sigma, Burlington, MA) for 10 minutes and adjusted to 2% w/v N-lauroylsarcosine and incubate for 10 minutes on ice. Samples are then centrifuged at 100,000 x g for 1 hour at 4°C and the supernatant discarded. The pellet is resuspended in 0.1% w/v sarkosyl solution in DPBS and stored at -80°C.

### Protein misfolding cyclic amplification

PMCA was performed as described previously [63]. Briefly, samples treated either with PK or with 0M, 2M and 4M Gdn-HCl are diluted to a 1:10 ratio in uninfected hamster brain homogenized to 10% w/v in PMCA conversion buffer (phosphate-buffered saline [pH 7.4] containing 6 mM EDTA [Millipore Sigma, Burlington, MA], 150 mM NaCl [Millipore Sigma, Burlington, MA], 100 μg/mL Heparin [Millipore Sigma, Burlington, MA], 0.05% [w/v] Digitonin [Millipore Sigma, Burlington, MA[, 1% [v/v] Triton X-100 [Millipore Sigma, Burlington, MA], and complete protease inhibitor cocktail [Millipore Sigma, Burlington, MA]). Samples were loaded into a QSonica Q700MPX sonicator (Newtown, CT) and subjected to cycles of 1 second sonication and 10 minutes incubation at 37°C for 72 hours. The sonicated samples were diluted to a 1:10 ratio in fresh uninfected hamster brain homogenate and subjected to another round of PMCA. Following PMCA, PrP^Sc^ was detected via Western blot as described below.

### SDS-PAGE and Western blot

Western blot analysis of these samples is performed as previously described [64]. Briefly, a 2:1 ratio of sample to 4x sample buffer (8% w/v SDS [ThermoFisher; Waltham, MA], 4% v/v b-mercaptoethanol [Millipore Sigma, Burlington, MA], 40% v/v glycerol [ThermoFisher; Waltham, MA], 0.004% w/v Bromophenol blue [Millipore Sigma, Burlington, MA], in 0.5 M Tris buffer, pH 6.8), boiled at 100°C for 10 minutes and size fractionated on 4–12% Bis-Tris NuPAGE polyacrylamide gel (Invitrogen, Carlsbad, CA), and transferred to a polyvinylidene difluoride (PVDF) membrane (Immobilon FL; Millipore Sigma, Burlington, MA). The membrane was blocked with 5% w/v nonfat dry milk (BioRad Laboratories, Hercules, CA) in 0.05% v/v tween tris-buffered saline (TTBS, BioRad Laboratories, Hercules, CA) for 30 minutes and the hamster prion protein detected by the mouse monoclonal anti-PrP antibody 3F4 (final concentration of 0.1 μg/mL, Millipore Sigma, Burlington, MA) or 12B2 (final concentration of 0.2 μg/mL, Wageningen Bioveterinary Research, Wageningen, The Netherlands). Western blots were developed using Pierce SuperSignal West Femto maximum-sensitivity substrate per manufacturer’s instructions (Pierce, Rockford, IL) and imaged on a Li-Cor Odyssey Fc Imager (Li-Cor, Lincoln, NE).

## Supporting information

S1 Fig Immunoreactivity of anti-PrP monoclonal antibodies 12B2 and 3F4 for HY and DY PrP^Sc^. (A) Diagram of the PrP protein (green line) and the different PK cleavage sites (red triangle) for HY and DY PrP^Sc^ which results in digestion of the N-terminus (blue line). The 3F4 epitope is present on both strains following PK digestion, while 12B2 is present only on HY PrP^Sc^. (B) Representative Western blot analysis of prion infected brain homogenate shows differential detection between 3F4 and the strain-specific antibody 12B2 at their respective antibody dilutions. (C) Representative Western blot analysis and (D) quantification of PrPSc abundance of serial 2-fold dilution of HY TME-infected brain homogenate probed with either the 3F4 or 12B2 anti-PrP antibodies indicates similar sensitivities of HY PrP^Sc^ detection.
(TIFF)

S2 Fig Western blot analysis of PK digested brain homogenates from serial high titer passage of DY TME in hamsters. Brain homogenates from hamsters infected with DY TME at 10^−4^ dilution (DY Inoc.) or serial high titer passage by either the intracerebral (i.c.), intraperitoneal (i.p) or extranasal (e.n.) routes of inoculation were digested with proteinase K prior to Western blot analysis. Western blots were probed with either the monoclonal anti-PrP antibodies 3F4 or 12B2.
(TIFF)

S3 Fig Extended PK digestion of HY or DY TME-infected brain homogenates reveals strain-specific differences in PrP^Sc^ degradation. Western blot analysis of brain homogenates from either HY or DY TME-infected animals were incubated with PK ranging from 0–400 μg/ml for 24 hours at 37°C.
(TIFF)

S4 Fig Serial PMCA passage of DY TME retains properties of DY TME. First (panels A-D) and second (panels E-H) serial rounds of PMCA reactions seeded with either uninfected (panels A,C,E,G) or non-PK digested DY TME-infected brain (panels B,D,F,H) were analyzed by Western blot for the presence of PrP^Sc^ using either the 3F4 (panels A,B,E,F) or 12B2 (panels C,D,G,H) anti-PrP antibodies.
(TIFF)

S5 Fig Survival curves of hamsters infected with CSSA products from Table 1. A) Hamsters inoculated with CSSA reactions, inoculated with B) second hamster passage of 2M or 4M CSSA from panel A and C) third serial hamster passage of 4M CSSA products from panel B. Groups of mock-infected animals were included with each inoculum. Circles indicate an absence of clinical signs of prion disease, squares indicated clinical signs of progressive lethargy and triangles indicate clinical signs of hyperexcitability.
(TIFF)

S1 Table Incubation period, attack rate, clinical signs, and PrP^Sc^ properties of hamsters infected with CSSA reaction products. (DOCX)
