# Supplementary figures and images for: Evidence for preexisting prion substrain diversity in a biologically cloned prion strain
Source: PLoS Pathog. 2023 Sep 5;19(9):e1011632. doi: 10.1371/journal.ppat.1011632 (PMC10503715; doi:10.1371/journal.ppat.1011632)

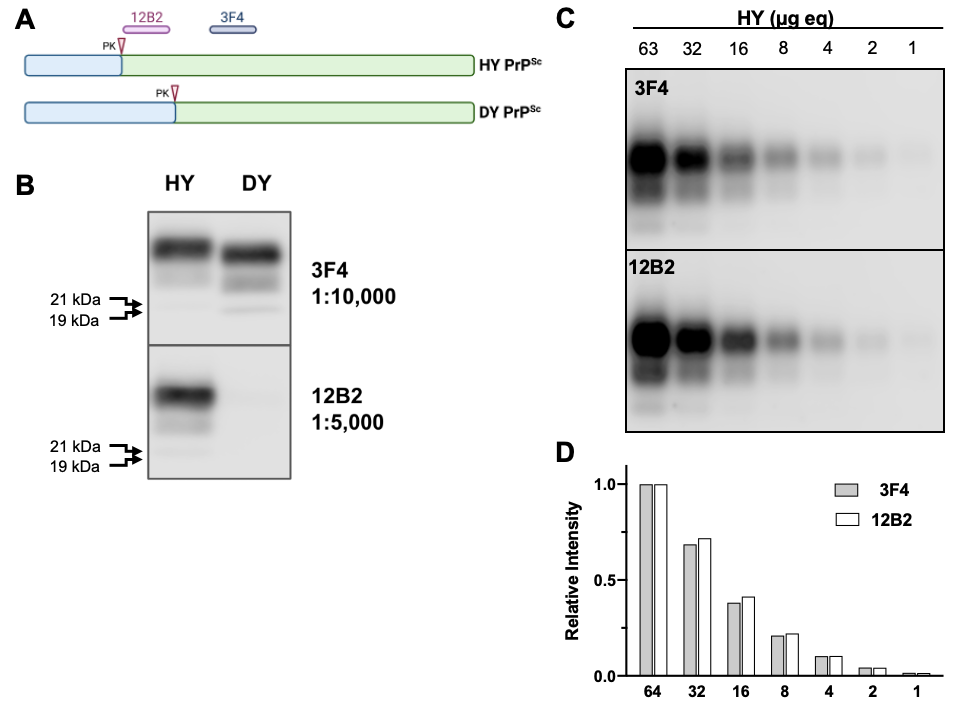

Supplement: S1 Fig — (A) Diagram of the PrP protein (green line) and the different PK cleavage sites (red triangle) for HY and DY PrPSc which results in digestion of the N-terminus (blue line). The 3F4 epitope is present on both strains following PK digestion, while 12B2 is present only on HY PrPSc. (B) Representative Western blot analysis of prion infected brain homogenate shows differential detection between 3F4 and the strain-specific antibody 12B2 at their respective antibody dilutions. (C) Representative Western blot analysis and (D) quantification of PrPSc abundance of serial 2-fold dilution of HY TME-infected brain homogenate probed with either the 3F4 or 12B2 anti-PrP antibodies indicates similar sensitivities of HY PrPSc detection. (TIFF) [file ppat.1011632.s001.tiff]

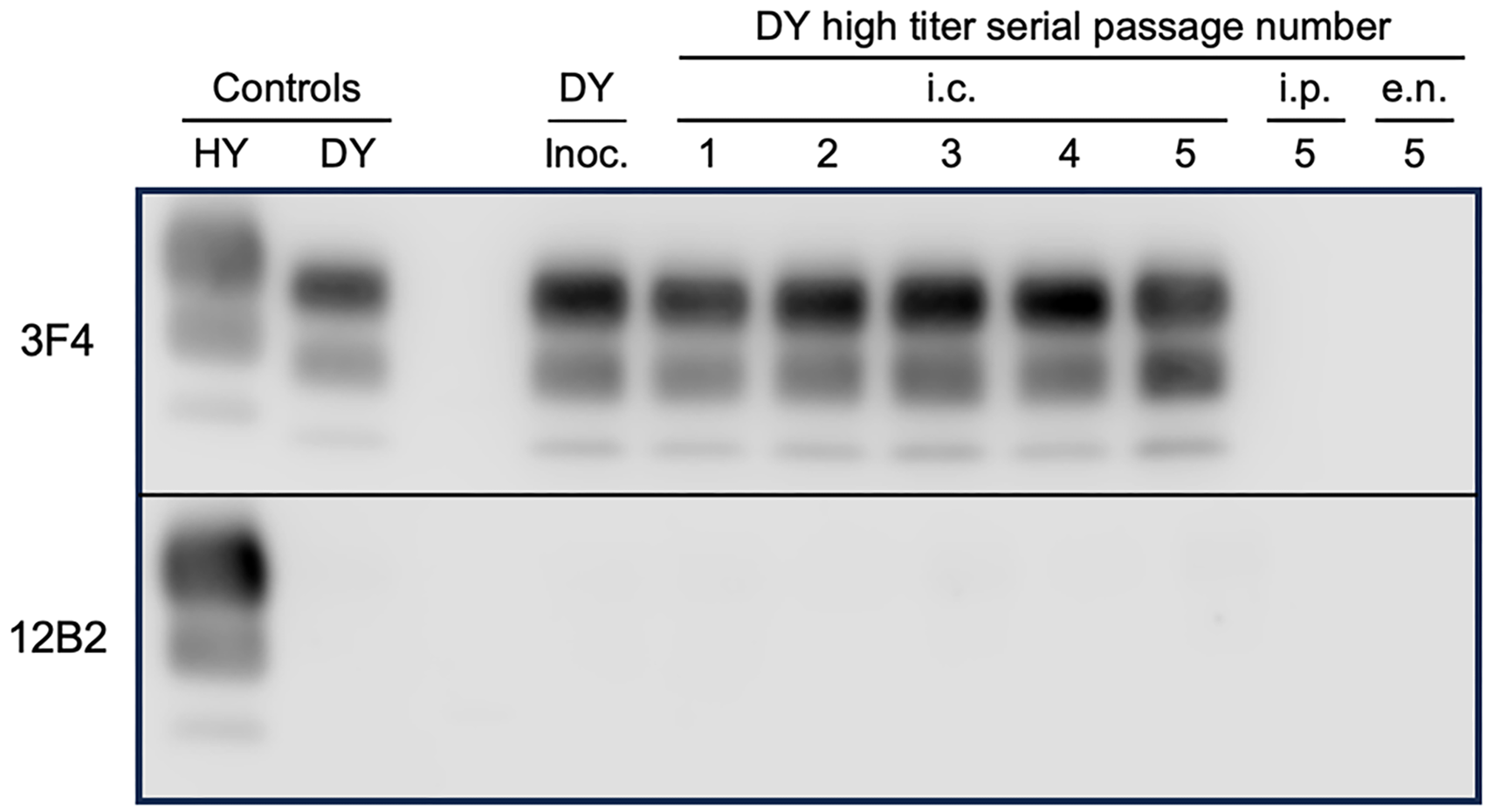

Supplement: S2 Fig — Brain homogenates from hamsters infected with DY TME at 10−4 dilution (DY Inoc.) or serial high titer passage by either the intracerebral (i.c.), intraperitoneal (i.p) or extranasal (e.n.) routes of inoculation were digested with proteinase K prior to Western blot analysis. Western blots were probed with either the monoclonal anti-PrP antibodies 3F4 or 12B2. (TIFF) [file ppat.1011632.s002.tiff]

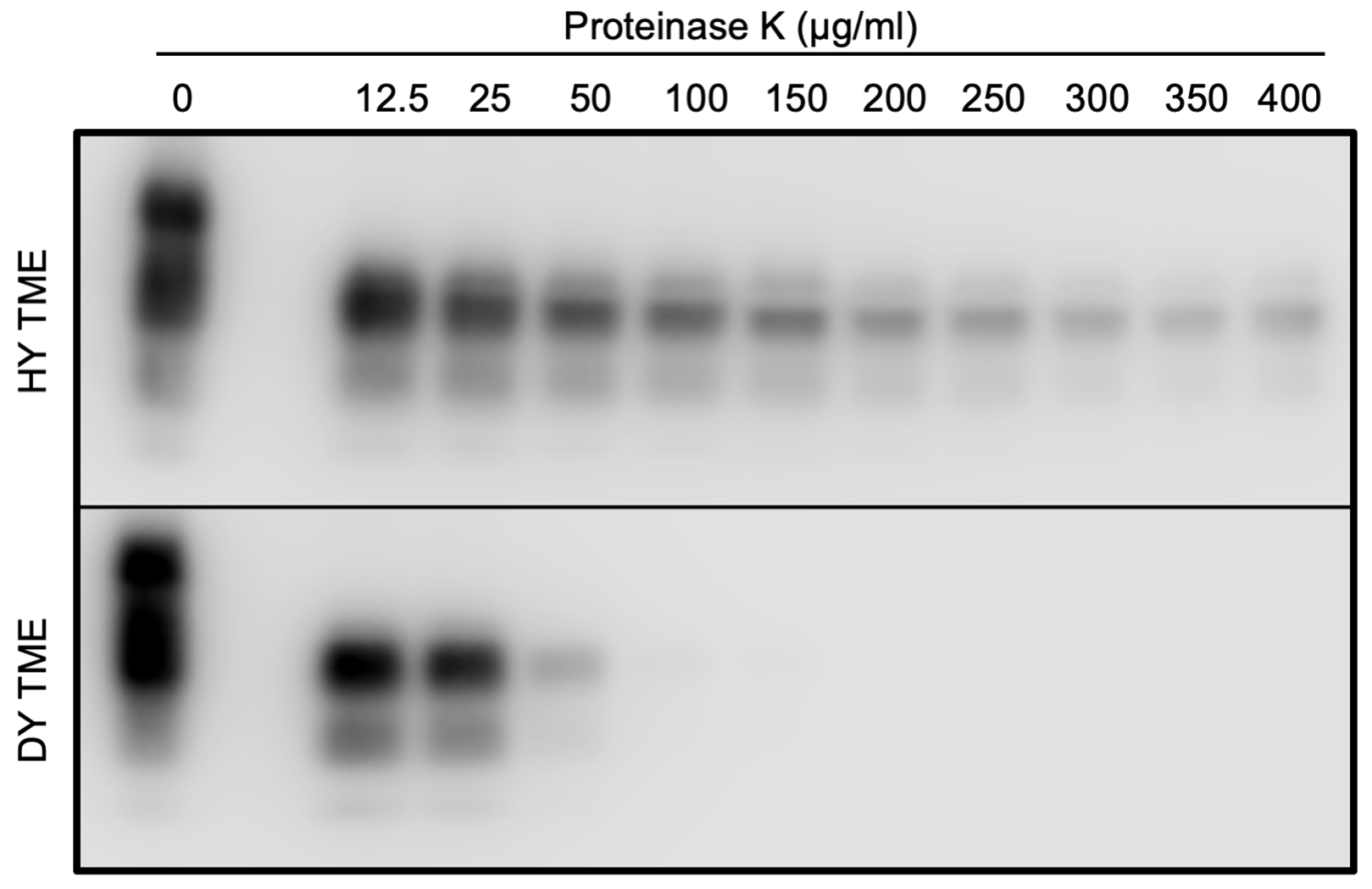

Supplement: S3 Fig — Western blot analysis of brain homogenates from either HY or DY TME-infected animals were incubated with PK ranging from 0–400 μg/ml for 24 hours at 37°C. (TIFF) [file ppat.1011632.s003.tiff]

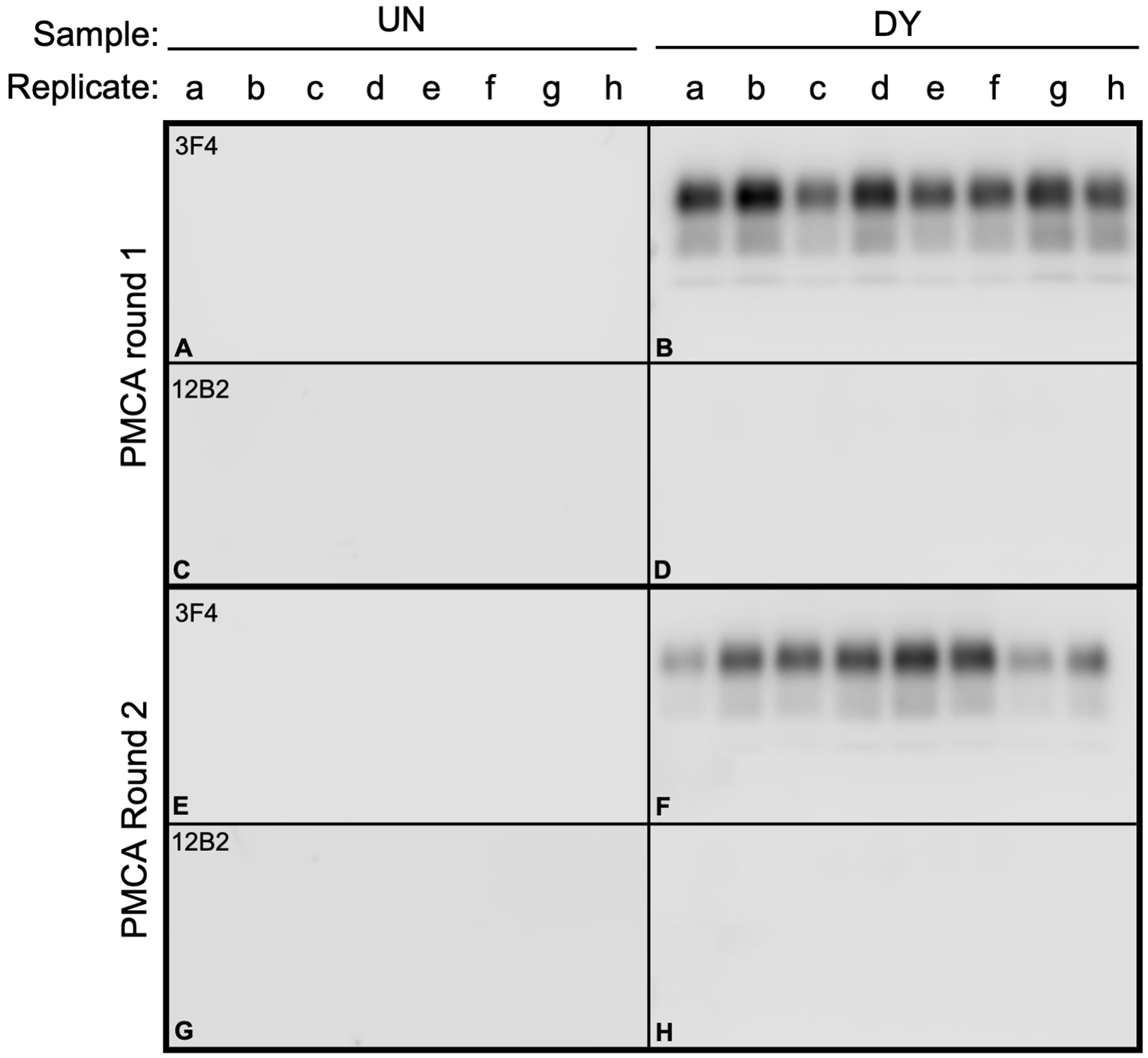

Supplement: S4 Fig — First (panels A-D) and second (panels E-H) serial rounds of PMCA reactions seeded with either uninfected (panels A,C,E,G) or non-PK digested DY TME-infected brain (panels B,D,F,H) were analyzed by Western blot for the presence of PrPSc using either the 3F4 (panels A,B,E,F) or 12B2 (panels C,D,G,H) anti-PrP antibodies. (TIFF) [file ppat.1011632.s004.tiff]

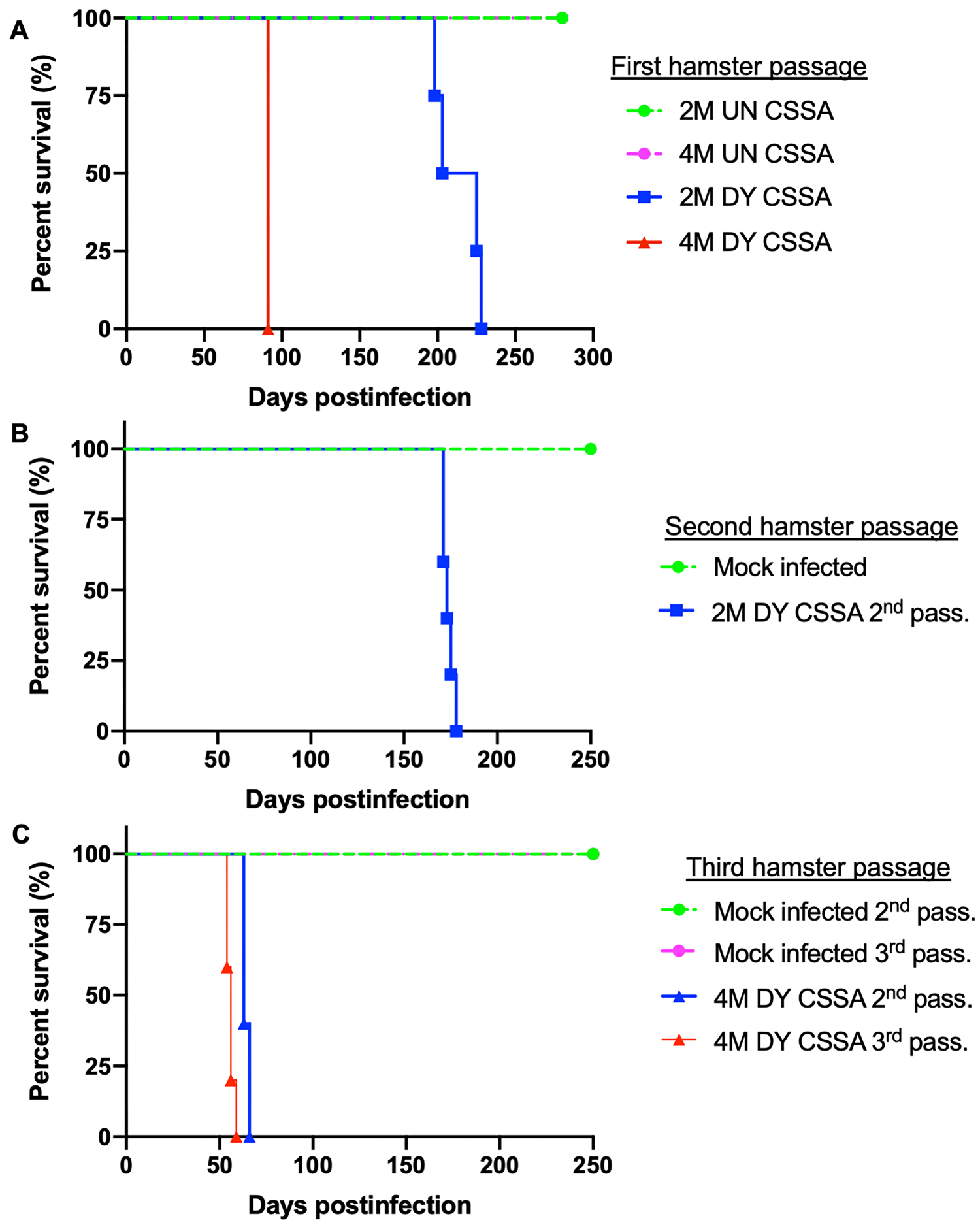

Supplement: S5 Fig — A) Hamsters inoculated with CSSA reactions, inoculated with B) second hamster passage of 2M or 4M CSSA from panel A and C) third serial hamster passage of 4M CSSA products from panel B. Groups of mock-infected animals were included with each inoculum. Circles indicate an absence of clinical signs of prion disease, squares indicated clinical signs of progressive lethargy and triangles indicate clinical signs of hyperexcitability. (TIFF) [file ppat.1011632.s005.tiff]
