# Supplementary material for: Evidence for preexisting prion substrain diversity in a biologically cloned prion strain
Source: PLoS Pathog. 2023 Sep 5;19(9):e1011632. doi: 10.1371/journal.ppat.1011632 (PMC10503715; doi:10.1371/journal.ppat.1011632)
Supplement: S1 Table — (DOCX) [file ppat.1011632.s006.docx]

**Table S1.** Incubation period, attack rate, clinical signs, and PrP^Sc^ properties of hamsters infected with CSSA reaction products.

|  |  |  |  | PrP^Sc^ properties | |
| --- | --- | --- | --- | --- | --- |
| Inoculum | Inc. period^a^ | Attack rate^b^ | Clinical signs | Migration | [Gdn-HCl]_1/2_ |
| *CSSA* |  |  |  |  |  |
| UN 2M | >280 | 0/5 | n.a | n.a. | n.a. |
| UN 4M | >280 | 0/5 | n.a | n.a. | n.a. |
| DY 2M | 214±3 | 4/5 | PL | 19 kDa | 2.05±0.04 (n=8) |
| DY 4M | 91±3 | 5/5 | H | 21 kDa | 2.46±0.07 (n=13) |
|  |  |  |  |  |  |
|  |  |  |  |  |  |
| *Brain derived prions* |  |  |  |  |  |
| DY 2M - 1^st^ Ha. pass. | 174±3 | 5/5 | PL | 19 kDa | 1.94±0.02 (n=16) |
| Mock | >250 | 0/5 | n.a | n.a. | n.a. |
|  |  |  |  |  |  |
|  |  |  |  |  |  |
| DY 4M - 1^st^ Ha. pass. | 65±3 | 5/5 | H | 21 kDa | 2.57±0.03 (n=30) |
| Mock | >250 | 0/5 | n.a | n.a. | n.a. |
|  |  |  |  |  |  |
| DY 4M - 2^nd^ Ha. pass. | 59±3 | 5/5 | H | 21 kDa | 2.34±0.04 (n=16) |
| Mock | >250 | 0/5 | n.a | n.a. | n.a. |
|  |  |  |  |  |  |
|  |  |  |  |  |  |
| HY TME | 60±3 | 5/5 | HA | 21 kDa | 2.33±0.02 (n=16) |
| DY TME | 169±4 | 5/5 | PL | 19 kDa | 1.95±0.01 (n=34) |
| Mock | >225 | 0/5 | n.a. | n.a. | n.a. |
|  |  |  |  |  |  |

^a^ days post infection±SEM

^b^ number inoculated / number affected

n.a. – not applicable

CSSA – Conformational stability and selection assay

UN – Uninfected

DY – DY TME

PL – progressive lethargy

H – hyperexcitability
